# Spectro-Fluor™ Technology for Reliable Detection of Proteins and Biomarkers of Disease: A Pioneered Research Study

**DOI:** 10.3390/diagnostics4040140

**Published:** 2014-09-29

**Authors:** Farid Menaa, Bouzid Menaa, Olga N. Sharts

**Affiliations:** 1 Department of Engineering and Biomedical Technology, Fluorotronics USA, Inc. San Diego, CA 92081, USA; E-Mail: olgasharts@gmail.com; 2 HYMETEC SA, Infection Control, Isnes 5032, Belgium; E-Mail: bouzid.menaa@gmail.com

**Keywords:** Spectro-Fluor™; Carbon-Fluorine spectroscopy, biophotonics, analytical laser-based instrumentation, C-F bond, Protein characterization, biomarkers of disease, cancer cells, *in-vitro* diagnostics

## Abstract

Quantitative and qualitative characterization of fluorinated molecules represents an important task. Fluorine-based medicinal chemistry is a fast-growing research area due to the positive impact of fluorine in drug discovery, and clinical and molecular imaging (e.g., magnetic resonance imaging, positron emission tomography). Common detection methods include fluorinated-based labelling using radioactive isotopes or fluorescent dyes. Nevertheless, these molecular imaging methods can be harmful for health due to the potential instability of fluorochromes and cytoxicity of radioisotopes. Therefore, these methods often require expensive precautionary measures. In this context, we have developed, validated and patented carbon-fluorine spectroscopy (CFS™), recently renamed Spectro-Fluor™ technology, which among a non-competitive family of in-house made devices called PLIRFA™ (Pulsed Laser Isochronic Raman and Fluorescence Apparatus™), allows reliable detection of Carbon-Fluorine (C-F) bonds. C-F bonds are known to be stable and safe labels once incorporated to any type of molecules, cells, compounds or (nano-) materials. In this pioneered research study, we used Spectro-Fluor™ to assess biomarkers. As a proof-of-principle experiment, we have established a three-step protocol intended to rapid protein detection, which simply consisted of: (i) incorporating a sufficient concentration of an aromatic amino-acid (fluorinated *versus* non-fluorinated) into cultured cells; (ii) simultaneously isolating the fluorinated protein of interest and the non-fluorinated form of the protein (control) by immune-precipitation; (iii) comparatively analyzing the respective spectrum obtained for the two protein forms by Spectro-Fluor™. Thereby, we were able to differentiate, from colon cancer cells HCT-116, the fluorinated and non-fluorinated forms of p21, a key transcriptional factor and downstream target of p53, the so-called “guardian of the genome”. Taken together, our data again demonstrates the beneficial alternative use of Spectro-Fluor™, which once combined with an innovative methodology permits one to quickly, reliably, safely and cost-effectively detect physiological or pathological proteins in cells.

## 1. Introduction

**Figure 1:**
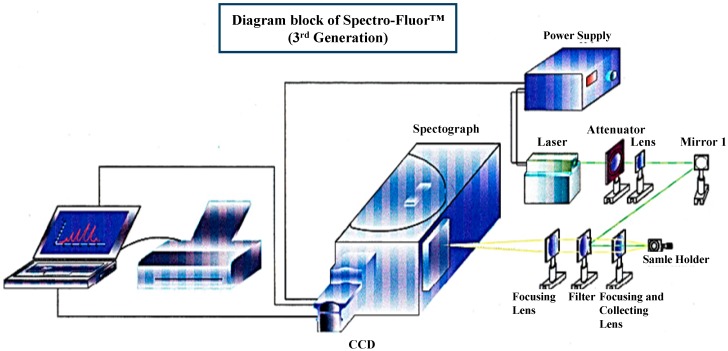
3D-diagram of the Spectro-Fluor™ technology. This patented apparatus represents the third generation of the device, and includes a laser with power unit, a spectrograph, an ICCD camera with image intensifier, a desktop computer with appropriate software for data analysis. The next generation will represent a compact and portable device with focusing lenses replaced by fiber optics.

The concept of Spectro-Fluor™ technology is mainly based on the discovery of characteristic C-F fingerprint(s) in the spectral area of 500–900 cm^−1^ allowing detection, characterization, imaging, monitoring, screening and measurement of fluoro-nanomaterials, fluoro-compounds, fluoro-molecules, fluoroorganic impurities or fluoro-degradation products [1,2,3]. Spectro-Fluor™ *aka* Carbon-Fluorine Spectroscopy™ (CFS™) is one of the key tools of our in-house technological platform called PLIRFA™ (Pulsed Laser Isochronic Raman and Fluorescence Apparatus™), which provides alternative solutions and applications across the biomedical (e.g., molecular/cell/tissue imaging), life (e.g., unravelling of molecular interactions, structures, conformations), pharmaceutical (e.g., drug design, delivery, tracing, discovery), environmental (e.g., characterization of pesticides, other contaminants), and nano(bio)technological (e.g., characterization of nano-molecules/materials) fields [1,2]. Thereby, Spectro-Fluor™ represents an innovative, disruptive, green, affordable, cost-effective, reliable and flexible analytical tool of high resolution, sensitivity and specificity (Figure 1) [1,2,3]. Interestingly, this technology constitutes a major advance over conventional spectroscopy technologies (e.g., conventional Raman tools) because of its capability to: (i) highly specific and ultrasensitive detect (ppm-ppb level or <10 cells) C-F bonds (–C-F, =CF_2_, –CF_3_); (ii) detect and characterize any halo-organic bonds or unlabeled molecules, regardless their physical state (solid, liquid or gas), via glass and polymer containers, quartz vials; (iii) acquire data in real-time (0.1 second per data point from 1000 average pulses); (iv) quantitatively determine any fluorinated substance since the emitted C-F bond(s) signal is directly proportional to the analyte concentration; (v) unravel chemical structures (e.g., molecular length determination, molecular analogs differentiation with as low as one carbon resolution); (vi) preserve the sample integrity (*i.e.*, non-destructive and non-invasive technology) allowing re-using of the sample; (vii) analyze any unprepared sample in a solvent-less/green fashion; (viii) easily provide data interpretation thanks to its interconnection with powerful software and in-house developed databases; (ix) be used with low maintenance/calibration requirements; (x) operate using adequate time-gating and time-delays with a single laser source functioning in three modes: continuous detection, pulsed detection and two photons excitation/time resolved fluorescence; (xi) be conveniently employed anywhere due to new generation of portable/compact devices incorporating fiber optics; (xii) be versatile, flexible, progressive and hyphenated opening possibilities to be coupled/hybridized to diverse technology platforms such as high-throughput screening (HTS) (e.g., arrays, multiplex assays systems), microscopy and other imaging systems (e.g., confocal laser scanning microscopy, atomic force microscopy (AFM), positron emission tomography (PET), magnetic resonance imaging (MRI), computed tomography (CT)), chromatography (e.g., high-performance liquid chromatography (HPLC), mass spectrometry (MS)), sorting instruments (e.g., fluorescence-activated cell sorting (FACS) or magnetic-activated cell sorting (MACS)).

Besides, the specific features of fluorine in the formation of fluorocarbons make it attractive in the design of non-viscous but polar organic compounds, with a polarity limited to influencing the intra-molecular nature and inter-molecular interactions with the microenvironment [4,5]. Interestingly, carbon-fluorination displays the additional following properties [6,7]: (i) enhancement of thermal stability (10^7^ Kcal/mol); (ii) increase of lipophilicity, hydrophobicity and solubility; (iii) improvement of the molecular bioavailability (e.g., diminution of the basicity of neighboring amines); and (iv) size comparable to H (*i.e*., 1.47 Å *versus* 1.20 Å) capable of mimicking enzyme substrates. Importantly, C-F bonds are [1,2,3,4]: (i) unique in nature; (ii) smaller and more stable than fluorescent dyes due to their covalent interactions; (iii) much less toxic than radioisotopes avoiding handling and logistic problems; (iv) less harmful at long-term exposure than radio-waves; and (v) inexpensively incorporated into molecules, compounds, materials or cells. Therefore, C-F bond(s) can be used in a number of industrial and academic areas including pharmacy, medicine and nanotechnology to characterize them, monitor them or confer some enhanced physicochemical features [1,2,3,4]. In terms of applications, a C-F bond can be employed as [1,2,3,4,8,9,10,11]: (i) a pharmaceutical security label to enhance the drug safety, thereby preventing and banning illegitimate drugs (*i.e.*, counterfeited or sub-standard drugs); (ii) a nano-label to enable drug trace during the whole pharmaceutical development process (*i.e.*, discovery, synthesis and production cycles, clinical and post-approval stages), subsequently bringing the product faster to the market; (iii) an *ex vivo*, *in situ* or *in vivo* label for biologics (e.g., peptides, proteins including antibodies, nucleic acids) and cells/tissues (e.g., stem cells, tumor cells); (iv) a functional chemical group for nanomaterials (e.g., nanoparticles, nanocarbon tubes, nanogels, nanoemulsions, nanoporous silica glasses).

Nowadays, although several techniques and technologies are employed to detect and characterize proteins and biomarkers of disease are available in the market (e.g., enzyme-linked immunosorbent assay (ELISA), Western blot), we propose here an original molecular method to quickly, reliably and cost-effectively detect a protein of interest (e.g., biomarker of disease) from cancer cells using the unique Spectro-Fluor™ biotechnology.

## 2. Materials and Methods

### 2.1. Presentation of Spectro-Fluor™ Technology

Spectro-Fluor™ technology (*aka* CFS™) is a key member of the progressive PLIRFA™ (Pulsed Laser Isochronic Raman and Fluorescence Apparatus™) technology platform, which is associated with a panel of patented methods and applications. The apparatus has been assembled onsite by our engineers and Raman experts in collaboration with other partners, manufacturers and providers worldwide. The design, concept and mode of functioning of this original technology have been previously described [1]. Briefly, the Spectro-Fluor™ device is composed of a metal—usually a copper or gold—vapor laser integrated with a power unit (Sierra, General Atomics, San Diego, CA, USA), a spectrophotometer Shamrock SR-303i integrated with a power supply ELC-00376 (Andor, Belfast, Northern Ireland), an ICCD camera DH-740-18F-33 including an image intensifier/photomultiplier (Andor, Belfast, Northern Ireland), an optical filter RazorEdge^®^ LP03-532RU-25 (Semrock Inc., Rochester, NY, USA), a computer and a software such as the software-time resolved Solis(t) (Andor, Northern Ireland) which was used in this study for data collection and processing. The global experimental setup is shown in the Figure 1.

### 2.2. Cells, Cells Culture and Cells Treatment

Human colon carcinoma HCT-116 cells (ATCC^®^ CCL-247™) were maintained at 37 °C in a 5% CO_2_ incubator, and cultured in McCoy’s 5a medium (Invitrogen) supplemented with 10% Fetal Bovine Serum (IBL, Maebashi, Japan), 1.5 mM l-Glutamine (Gibco BRL, Rockville, MD), 100 units/mL Penicillin (Gibco BRL), 100 μg/mL Streptomycin (Gibco BRL). 5.10^6^ cells, cultured in 100 mm dish containing 4 mL of cell culture medium, were then treated with 10 mg/mL of fluorinated amino-acid (*i.e.*, 4-fluoro-l-phenylalanine) (Acros) or 10 mg/mL of the corresponding fluorinated-free amino-acid (*i.e.*, l-phenylalanine) (Acros) used as quality control. In order to minimize the physiological degradation of the endogenous p21 protein, which is already barely detectable at unstressed conditions, 50 μM of the potent proteasome inhibitor LLnL (Sigma-Aldrich, St. Louis, MO) dissolved in dimethylsulfoxide (DMSO) was meantime added to the cells. The cells were harvested immediately after addition of phenylalanine residues into medium-containing cells (time-dependent control) and 24 h later, which represents sufficient doubling time for HCT-116 cells to efficiently incorporate amino-acids, Three independent experiments have been performed.

### 2.3. Enrichment of Purified Protein by Immune-Precipitation

All the cells were collected, homogenized and carefully counted. Cells treated with fluorinated phenylalanine (F-Phe) were not mixed with non-fluorinated phenylalanine (Phe) treated cells, and the whole purification process was done in the same experimental conditions. About 5.10^6^ cells were washed twice with 1× PBS and re-suspended in 200 μL RIPA buffer (50 mM Tris-HCl [pH 8.0], 100 mM NaCl, 5 mM EDTA, 10 μM NaF, 1 mM Na_3_VO_4_, 1 μM Okadaic Acid, 2 mM DTT, 0.25% NP-40 and 0.5 mM PMSF). The lysates were then sonicated and clarified by centrifugation at 15,000× *g* for 30 min. The respective subsequent supernatant was collected and incubated with a goat anti-p21 antibody (Santa Cruz Biotechnology, Dallas, TX, USA) for immunoprecipitation at 4 °C during 1.5 h. 20 μL of protein A sepharose beads (Amersham Pharmacia Biotech Inc, Piscataway, NJ, USA) were added and further incubated for 1 h. The beads were washed five times with 1 mL of RIPA buffer and the proteins were recovered by boiling. The p21 proteins spectra were ultimately analyzed by Spectro-Fluor™ (Fluorotronics Inc, San Diego, CA, USA).

### 2.4. Protein Analysis by Spectro-Fluor™ Technology

Both the F-p21 and the endo-exogenous p21 proteins were analyzed by Spectro-Fluor™ Technology (Fluorotronics Inc, San Diego, CA, USA). 500 μL of the respective samples were analyzed in standard vials. The experimental setup is shown in Figure 1. A 532 nm DPSS Q-switched laser (Sierra, General Atomics, San Diego, CA, USA) with 20 kHz repetition rate and 10 ns pulse duration was used to deliver as small as 10 mW of average power to the sample. The lens focused the beam into the given sample and collected the scattered radiation back along the same axis at 570 nm. The data were collected from three independent experiments and processed using a computer and the software-time resolved Solis(t) (Andor, Northern Ireland).

## 3. Results and Discussion

**Figure 2:**
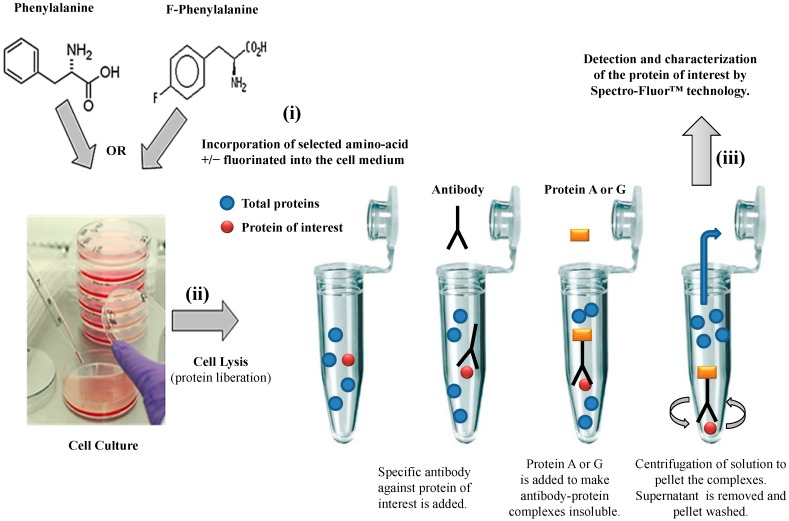
Flow chart of the experimental methodology. The three-step process is resumed by: (**i**) *Ex-vivo* protein labeling by incorporation of either 4-fluoro-l-phenylalanine. l-phenylalanine is used as a control; (**ii**) immune-precipitation by using only one antibody against the antigen/epitope of the protein of interest; (**iii**) detection and characterization of the protein of interest by Spectro-Fluor™ technology.

We have developed an innovative photonic biotool called Spectro-Fluor™ Technology (Fluorotronics, Inc.) as well as a new *in vitro* method based on *ex vivo* incorporation of fluorinated amino acids into cells in order to detect lowly-expressed molecules such as biomarkers of disease. (Figure 1 and Figure 2). Indeed, the advantages of Spectro-Fluor™ Technology (*aka* Carbon-Fluorine spectroscopy) over routinely used laboratory methods (e.g., Western blots, ELISA, protein dot blots) are represented by the rapid, safe, reliable, highly sensitive and specifique detection of C-F bond(s) formed into molecules [1].

We have established a valuable *ex vivo* protein labeling protocol that consisted of three steps (Figure 2): (i) addition in excess (e.g., in this study, 10 mg/mL as non-limiting concentration) of a selected aromatic fluoro-aminoacid (e.g., in this study, 4-fluoro-l-phenylalanine) into an adequate medium-containing cells (e.g., in this study, human colon carcinoma HCT-116 cells). In parallel, the corresponding non-modified amino-acid (e.g., in this study, l-phenylalanine) was used as control; (ii) immediately after the amino-acid addition (control) and after a convenient period of time (e.g., in this study, 24 h), simultaneous isolation of both F-p21 and p21 (control) was performed by immune-precipitation (IP). IP is a procedure by which proteins or peptides that react specifically with an antibody are removed from solution and examined for quantity or physical characteristics. The expression of the two p21 protein forms (*i.e.*, F-p21 and p21) was routinely checked by Western blot, a widely used biochemical technique albeit long and expensive (data not shown due to low basal expression detectable with Western blot); (iii) comparative examination of the respective spectra obtained by Spectro-Fluor™ technology obtained in same experimental conditions.

This protocol could have been reduced in two steps (*i.e.*, steps 2 and 3) if a fluorinated antibody against our protein of interest was available at the time of our experiments (pending patent).

**Figure 3:**
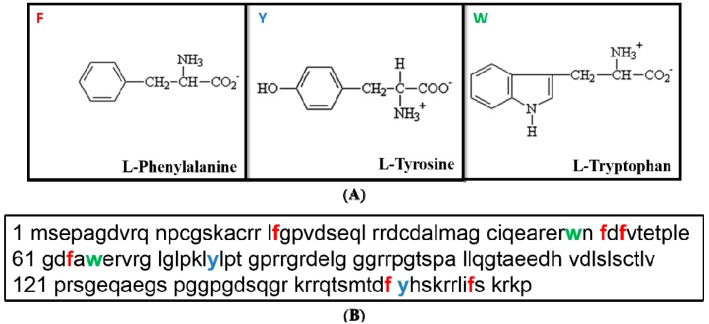
Tertiary sequence of p21. (**A**) Structure of the three aromatic acids, *i.e.*, Phe, Tyr and Trp. Phe contains only hydrophobic group whereas Tyr and Trp have both hydrophobic and hydrophilic side groups. (**B**) Relative frequency of the three aromatic amino acids in the p21 primary structure, *i.e.*, Phenylalanine/Phe/F (in red) is at number of 6, Tyrosine/Tyr/Y (in blue) and Tryptophane/Trp/W (in green) are at number of three [12].

The reasons why we opted for the phenylanine (Phe/F) aminoacid are the following: (i) l-Phe is an aromatic aminoacid, and it is known that molecules containing aromatic rings better scatter the light [1]; (ii) l-Phe is present twice more (*n* = 6) in the p21 sequence compared to the two other aromatic aminoacids which are tyrosine (Tyr/Y) and tryptophane (Trp/W) at the number of three (Figure 3A,B and Table 1). Even so, we also realized a parallel work with tyrosine (*i.e.*, 5-fluoro-dl-tyrosine) or and tryptophane (*i.e.*, 5-fluoro-dl-tryptophane), which led to same conclusions, albeit the best Spectro-Fluor™ signal was obtained with phenylalanine (data not shown).

**Table 1:** Single-letter amino-acid code, In accordance to the international genetic system.

Full Name of Amino-Acid	Single-Letter Code	Three-Letters Code
Glycine	G	Gly
Alanine	A	Ala
Leucine	L	Leu
Methionine	M	Met
Phenylalanine	F	Phe
Tryptophan	W	Trp
Lysine	K	Lys
Glutamine	Q	Gln
Glutamic Acid	E	Glu
Serine	S	Ser
Proline	P	Pro
Valine	V	Val
Isoleucine	I	Ile
Cysteine	C	Cys
Tyrosine	Y	Tyr
Histidine	H	His
Arginine	R	Arg
Asparagine	N	Asn
Aspartic Acid	D	Asp
Threonine	T	Thr

The choice of the p21 protein (*aka* WAF1, CAP20, Cip1 or Sdi1) is explained by the important following facts: (i) p21 is a key transcriptional factor, a cyclin kinase inhibitor (CKI), and a downstream target of p53, the so-called “guardian of the genome” potentially acting as an immune-suppressor. Thereby, p21 elicits pleiotropic biological functions of major biomedical interest (e.g., cell cycle arrest, gene expression regulation, DNA replication, DNA response to DNA damage, p53-dependent or -independent apoptosis (*aka* programmed cell death), tumor suppression) [13,14,15,16,17,18,19,20,21]; (ii) p21 is a small protein (21 kDa, 164 base pairs) and its basal expression under unstressed conditions is barely detectable, rending its characterization difficult albeit certain genotoxic conditions, such as ionizing radiation, can be a good way to see it up-regulated [21]. In this regard, Spectro-Fluor™ technology, along with our molecular approach, constitutes a robust biotechnology to qualitatively and quantitatively determine p21. Further, the whole process is cost-effectively notably due to: (i) the use of only one antibody; (ii) the possibility to re-use the samples; (iii) the capability of Spectro-Fluor™ technology to reliably confirm the tissue-specific presence of p21 when coupled to conventional or advanced imaging systems.

**Figure 4:**
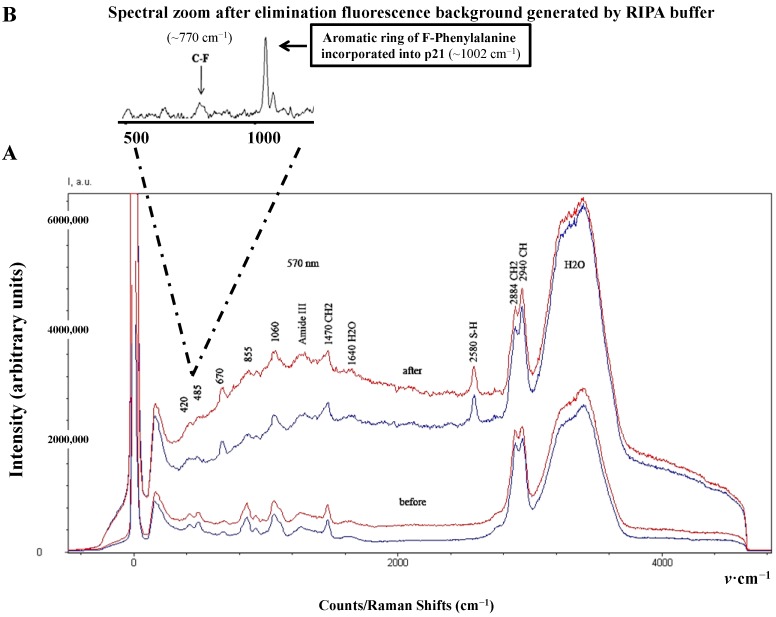
Spectro-Fluor™ spectra of the purified and enriched protein of interest. (**A**) Spectra in RIPA buffer of immune-precipitated p21 protein forms “before” (Time 0) and after (Time 24 h) effective incorporation of F-Phe (red spectrum) or non-labeled Phe (blue spectrum). 10 mg/mL of either amino-acid was added into McCoy’s 5a medium-containing HCT-116 colon cancer cells. (**B**) The F-p21 protein detection was performed after several washes with PBS 1X to significantly reduce the background generated by the RIPA buffer. The C-F signal is detected around 770 cm^−1^ and the aromatic bond optical assignment of the incorporated phenylalanine is expectedly observed at 1002 cm^−1^.

**Figure 5:**
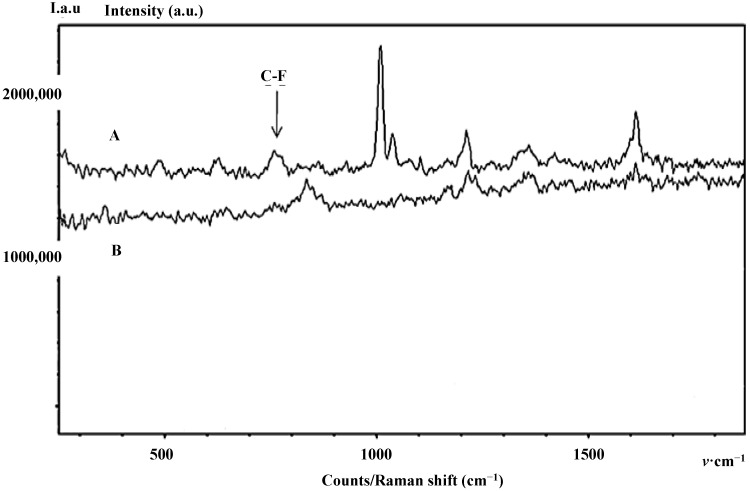
Background-Free Spectro-Fluor™ spectra of the purified and enriched protein of interest. Spectra in RIPA buffer of immune-precipitated p21 protein forms after (Time 24 h) effective incorporation of F-Phe (**A**) or non-labeled Phe. (**B**) 10 mg/mL of either amino-acid was added into McCoy’s 5a medium-containing HCT-116 colon cancer cells. In this experiment, the p21 protein forms detection was performed after several washes with PBS 1X to significantly reduce the background generated by the RIPA buffer. The C-F signal is detected around 770 cm^−1^ and the aromatic bond optical assignment of the incorporated F-Phe is expectedly observed at 1002 cm^−1^. It is interesting to note that, in our experimental conditions, the signal of F-Phe incorporated in p21 is very specific when compared with the control. Loss of sensitivity is certainly due to the few washings preformed to eliminate the fluorescence background generated by the RIPA buffer.

Interestingly, the introduction of fluorine, a chemical element with the highest electronegativity, is known to affect the optical property of amino acids in both frequency and intensity [4,22]. Thereby, the main vibrational assignments of F-p21 obtained by Spectro-Fluor™ are selectively represented by a weaker band between 635–670 cm^−1^, a C-F optical signature detected around 770 cm^−1^, and an intense band detected at 1002–1060 cm^−1^ including a slightly weaker band at 1039 cm^−1^ indicative of a mono-substituted benzene ring vibration due to successful incorporation of phenylalanine residues into p21 (Figure 4A,B, Figure 5 and Table 2).

**Table 2:** Main Spectro-Fluor™ vibrational assignments of the p21 protein.

Band (cm^-1^)	Vibrational mode	Mainly observed in:	References
670	C–S stretching	Cys	[22]
766	Symmetric ring breathing	Amino-acids, Proteins	[22]
855	Ring breathing	Tyr, DNA	[22]
925	C–C stretching	Proline, Valine, Protein	[22]
1060	C–C stretching	Lipids, Nucleic acids, Proteins	[22]
1250–1270	C–N stretch amide III	Proteins	[22]
1300	C–H_2_	Lipids, Proteins	[22]
1440–1470	CH_2_ deformation	Proteins	[22]
2580	S–H stretch	Thiol group of Cys, Methionine	[22]
2884	C–H_2_	Lipids	[22]
2940	C–H	Lipids, Proteins	[22]

Expectedly, similar and low spectrum intensity of both F-p21 and p21 were noticed after immediate incorporation of F-Phe or Phe into the medium-containing HCT-116 cells. In this context, the cells would not have sufficient time to incorporate the respective Phe aminoacid. Nevertheless, the difficult spectral discrimination at early time between the two p21 forms has been overcome after 24 h of cells culture. Indeed, the spectral intensity obtained with F-p21 was greater (*i.e.*, amplitude about 1.5×), compared to that of p21 (Figure 4A).

In order to eliminate the potential background due to two fluoride constituents in the RIPA buffer (*i.e.*, NaF (Sodium Fluoride), a phosphatase inhibitor and PMSF (PhenylMethylSulfonyl Fluoride), a protease inhibitor), which were required to preserve the protein integrity during immune-precipitation, we washed the samples several times with 1× PBS. Further, to avoid any misinterpretations regarding the sensitivity levels, three independent experiments for the four samples have been conducted in the same conditions. In agreement with our expectations, the amplitude of the signal intensity differed much when between F-p21 and p21 than between Y-p21 and p21 or between W-p21 and p21 (data not shown). This effect can be easily explained by the greater number of phenylalanine residues (*n* = 6) in the p21 protein sequence when compared with the equal twice-lower number of tyrosine and tryptophan residues (*n* = 3) (Figure 3B).

Taken together, our results are encouraging and strongly suggest that Spectro-Fluor™ technology could be reliably used for the detection and characterization of bioactive molecules. Although fluorination is a common process used to enhance properties of pharmaceutics [1], our present study pioneered the possibility to F-label proteins, including antibodies and biomarkers of diseases, which could be assessed by or used with Spectro-Fluor™ technology to reach a number of clinical objectives.

## 4. Conclusions

Fluorinated molecules, widely used in medicinal chemistry (e.g., drug design), emerge in the biomedical field (e.g., fluorinated bio-probes and theranostics™ such as F-nucleic acids, F-peptides) because of their special features (e.g., enhanced bioavailability and stability). Pharmacological studies suggest that fluorine modification may significantly reduce undesirable drug side-effects meantime offering superior efficacy. Therefore, we postulated that fluorinated proteins (e.g., biomarkers of disease, antibodies) should also be beneficial. Within the PLIRFA™ technology platform, which includes progressive analytical devices, we developed a becoming gold-standard Spectro-Fluor™ technology as well as a panel of green methods, enabling rapid, cost-effective, sensitive and specific detection and characterization of F-molecules, F-compounds, F-cells, F- tissues or F-(nano-)biomaterials. In this study, we successively show the possibility to detect a fluorinated protein (*i.e.*, p53-downstream target p21) by Spectro-Fluor™ technology. This strongly suggests that this innovative biotechnology could significantly offer alternative solutions for a wide number of applications (e.g., molecular design, molecular imaging, development of F-antibodies) and technologies routinely used in laboratories and hospitals (e.g., western-blot, enzyme-linked immune sorbent assay, mass-spectrometry, MRI, PET). Eventually, we report that Spectro-Fluor™ technology is able to reduce the troublesome detection associated with low-expressed molecules (e.g., p21).

## Abbreviations

AFM: Atomic Force Microscopy
C-F: Carbon-Fluorine
CFS: Carbone-Fluorine Spectroscopy
CT: Computed Tomography
DPSS: Diode-Pumped Solid-State
ELISA: Enzyme-Linked ImmunoSorbent Assay
FACS: Fluorescence-Activated Cell Sorting
HCT-116: Human colorectal carcinoma cell type
HPLC: High-Performance Liquid Chromatography
HTS: High-Throughput Screening
IP: Immunoprecipitation
MACS: Magnetic-Activated Cell Sorting
MRI: Magnetic Resonance Imaging
MS: Mass Spectrometry
NaF: Sodium Fluoride
PET: Positron Emission Tomography
Phe: Phenylalanine
PLIRFA: Pulsed Laser Isochronic Raman and Fluorescence Apparatus
PMSF: PhenylMethylSulfonyl Fluoride
R&D: Research and Development
RIPA: Radio-ImmunoPrecipitation Assay
Trp: Tryptophane
Tyr: Tyrosine
